# Gastric ulcer and duodenitis associated with coinfection of human herpesvirus-8 and cytomegalovirus in a renal transplant recipient: a case report

**DOI:** 10.1186/s12882-023-03127-z

**Published:** 2023-03-24

**Authors:** Sik Lee, Kyoung Min Kim, Hong Pil Hwang, Jeong-Hwan Hwang

**Affiliations:** 1 Department of Internal Medicine, Jeonbuk National University Hospital, 20 Geonji-Ro, Deokjin-Gu, 54907 Jeonju-Si, Republic of Korea; 2 Research Institute of Clinical Medicine of Jeonbuk National University, Jeonju, Jeonjuk Korea; 3 Biomedical Research Institute of Jeonbuk National University Hospital, Jeonju, Jeonbuk Korea; 4 Department of Pathology, Jeonbuk National University Hospital, Jeonju, Republic of Korea; 5 Department of Surgery, Jeonbuk National University Hospital, Jeonju, Republic of Korea

**Keywords:** CMV, HHV-8, Gastric ulcer, Duodenitis

## Abstract

**Background:**

The coinfection between cytomegalovirus (CMV) and either human herpesvirus‐6 (HHV‐6) or HHV-7 in renal transplant recipients is well known; however, there have been few reports of coinfection of CMV associated with HHV-8. This paper presents a first case of acute gastric ulcer and duodenitis associated with CMV and HHV‐8 coinfection after renal transplantation.

**Case presentation:**

A 33-year-old male with a history of kidney transplantation was admitted to hospital because of postural epigastric pain. The recipient was CMV seropositive prior to transplantation and received trimethoprim-sulfamethoxazole without universal prophylaxis. Approximately 5 months after renal transplant, the recipient complained postural epigastric pain. An endoscopy revealed diffuse ulcerative lesions in the lower body and in the antrum of the stomach, as well as several erythematous mucosal lesions in the duodenum. Histopathologic examination identified CMV inclusions consistent with invasive CMV disease and immunohistochemical staining showed positive results for HHV-8 and CMV. No tumorous diseases such as Kaposi’s sarcoma were detected. After 3 weeks of intravenous ganciclovir treatment, we observed that serum CMV PCR remained within the normal range and clinical symptoms improved. A follow-up endoscopy performed 3 weeks later showed that the severity of the above mentioned lesions had improved.

**Conclusions:**

We report the first case of a renal transplant recipient diagnosed with acute gastric ulcer and duodenitis associated with coinfection of CMV and HHV-8. Ganciclovir appears to be effective in diseases associated with coinfection of CMV and HHV-8.

## Background

Clinical manifestations of cytomegalovirus (CMV) infection in solid organ transplant recipients exhibit a wide range of effects from asymptomatic infection to CMV disease [1]. The development of tissue-invasive CMV disease varies according to the target organs involved, and the most common form is gastrointestinal disease which can affect any segment of the gastrointestinal tract [1]. CMV has been reported to have viral interactions through the coinfections with various viruses including human herpesvirus‐6 (HHV-6), HHV-7, Epstein-Barr virus (EBV), BK virus, human immunodeficiency virus (HIV), and hepatitis C virus (HCV) [2]. These viral interactions are one of the indirect effects of CMV infection.

HHV-8 infection causes Kaposi's sarcoma (KS) in AIDS patients and in organ transplant patients, and is associated with primary effusion lymphoma and multicentric Castleman’s disease [3]. Although it remains controversial the connection between HHV-8 infection and inflammatory disorders, HHV-8 has been reported to be associated with non-neoplastic diseases such as sarcoidosis, pemphigus, Kikuchi lymphadenopathy, hepatitis, bone marrow failure, lymphoproliferative B-cell disorders, and hemophagocytic lymphohistiocytosis [4, 5]. The cell tropism of HHV-8 coincides significantly with CMV cell tropism [6]. CMV has been detected in KS lesions of AIDS patients [7]. Some experimental data also suggest that CMV coinfection can induce HHV-8 lytic replication in a variety of cells including endothelial cells, fibroblasts, and keratinocytes [8]. An interaction between CMV and HHV-8 in vivo is likely to occur though this interaction has been rarely studied.

The coinfection between CMV and either HHV-6 or HHV-7 in renal transplant recipients is well documented; however, there have been few studies of CMV and HHV-8 coinfection in CMV diseases of renal transplant recipients. In this study, we report the first case of a renal transplant recipient diagnosed with acute gastric ulcer and duodenitis associated with coinfection of CMV and HHV-8.

## Case presentation

**Fig. 1:**
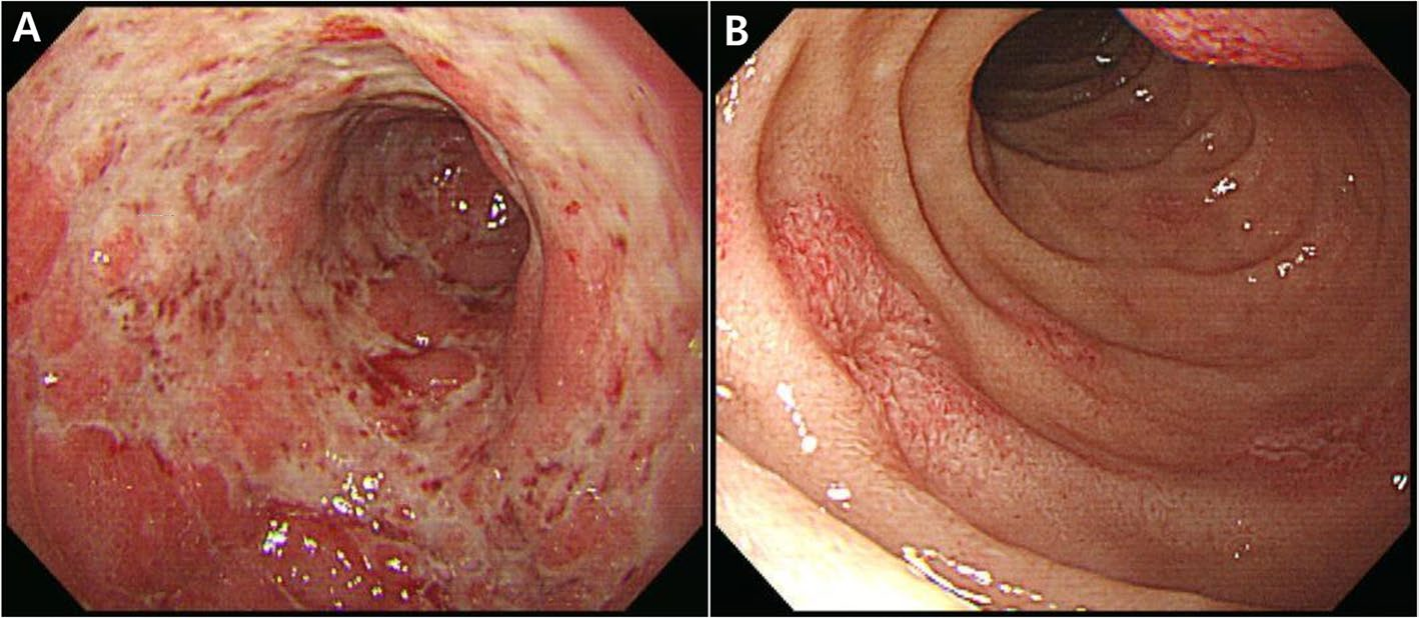
Upper endoscopic view. (**A**) Diffuse ulcerative lesions on the lower body and on the antrum of the stomach. (**B**) Erythematous mucosal lesions on the duodenum

**Fig. 2:**
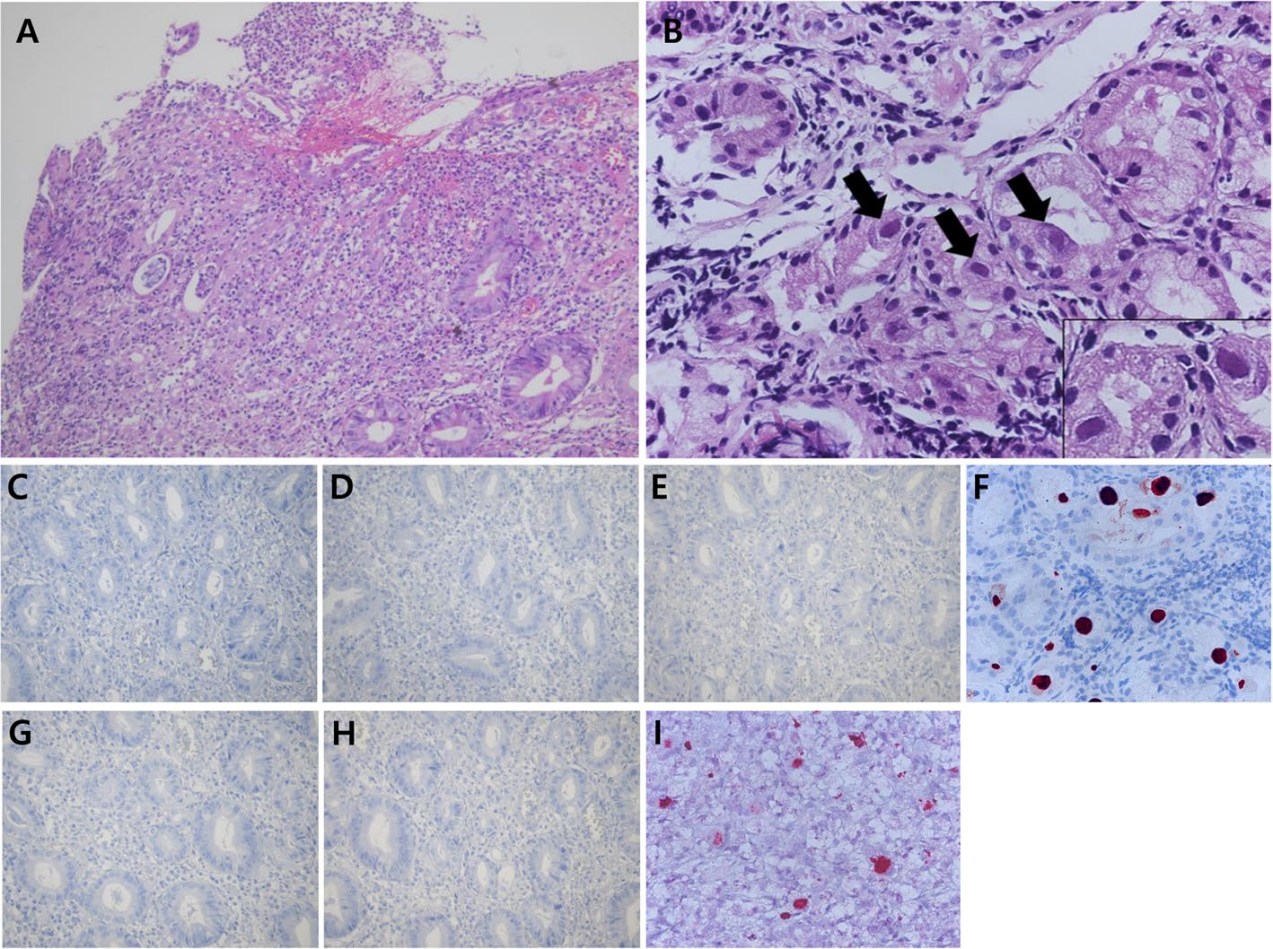
Histologic features of the biopsy. (**A**) Diffuse acute and chronic inflammatory cell infiltration in the ulcer of the stomach (H&E stain, original magnification: × 200). (**B**) At higher magnification, typical eosinophilic inclusion body (arrow and inset) in the nucleus of the cell in the lamina propria (H&E stain, original magnification: × 400). Immunohistochemical staining for herpes viruses. The biopsy was negative for (**C**) Herpes simplex virus (HSV)-1, (**D**) Varicella Zoster virus (VZV), and (**E**) Epstein-Barr virus (EBV). Immunohistochemical staining was positive for (**F**) cytomegalovirus (CMV) infected cells (original magnification: × 400). The biopsy was negative for (**G**) Human Herpesvirus (HHV)-6 and (**H**) HHV-7, and immunohistochemical staining for (**I**) HHV-8 was positive in the nucleus of infected cells (original magnification: × 400)

A 33-year-old male with a history of kidney transplantation was admitted to hospital because of postural epigastric pain, which began 3 days prior to admission. He also reported nausea, vomiting, and loss of appetite. His epigastric pain was reduced when lying supine and aggravated when sitting, standing, and ambulating. The patient’s epigastric pain was not relieved with acid suppressants. The patient had been diagnosed with gastroesophageal reflux after an endoscopy performed for chest discomfort one year prior to admission, for which he was provided with a proton pump inhibitor. Five months earlier, he had received a living donor kidney transplant for end-stage renal disease due to IgA nephropathy. He was treated with immunosuppressants including tacrolimus, mycophenolate mofetil, and prednisolone. He received basiliximab induction therapy at the time of transplantation and completed 6 months of trimethoprim-sulfamethoxazole treatment without universal antiviral prophylaxis following the renal transplant. No episodes of acute organ rejection occurred. His white blood cell count was 6.3 × 10^3^/μL, hemoglobin level was 11.0 g/dL, and platelet count was 2.2 × 10^5^/μL. His chemistry profile levels were as follows: total protein, 5.4 g/dL; albumin, 3.4 g/dL; blood urea nitrogen, 26 mg/dL; creatinine, 3.18 mg/dL; lactate dehydrogenase, 717 IU/L; C-reactive protein, 58.10 mg/L; and Tacrolimus, 5.72 ng/mL. To evaluate the patient’s epigastric pain, an upper endoscopy was performed, and the result showed diffuse ulcerative lesions on the lower body and on the antrum of the stomach, as well as several erythematous mucosal lesions in the duodenum (Fig. 1A and B). The result of a rapid urease test (CLO test) was negative. A histologic examination of the biopsy revealed ulcerative inflammation with diffuse infiltration of inflammatory cells (Fig. 2A). At high magnification, occasional tubular epithelial cells were observed to have eosinophilic inclusion bodies in the nuclei (Fig. 2B). The histologic findings suggested the possibility of viral infection, and immunohistochemical staining for herpesviruses were performed using Benchmark ULTRA, an automated immunohistochemistry stainer (Ventana Medical Systems Inc., Tucson, AZ, USA). The immunohistochemical staining was positive for CMV and HHV-8 (Fig. 2F and I), and negative for Herpes simplex virus-1, Varicella zoster virus, EBV, HHV-6, and HHV-7 (Fig. 2C, D, E, G, and H). A serologic test for CMV was CMV IgG-positive and CMV IgM-negative, and serologic tests for HIV, HBV, and HCV were negative. An HHV-8 serologic test was not performed. The result of a serum polymerase chain reaction (PCR) test for CMV was 1,090,000 copies/mL. The viral load of HHV-8 was not measured. Following a physical examination, radiologic tests, as well as pathologic analysis, the possibilities of Kaposi sarcoma, primary effusion lymphoma, and multicentric Castleman’s disease were excluded. Finally, the patient was diagnosed with acute gastric ulcer and duodenitis associated with a coinfection of CMV and HHV-8. After 3 weeks of treatment with intravenous ganciclovir, serum CMV PCR testing detected no viral copies, and clinical symptoms improved. A follow-up endoscopy performed 3 weeks later showed that the severity of the above-mentioned lesions had improved. During 3 years of follow-up assessments, no recurrence of gastric ulcer or duodenitis was observed.

## Discussion

In this study, we report the case of gastric ulcer and duodenitis associated with a coinfection of CMV and HHV-8 after renal transplantation. To our knowledge, this study is the first report concerning CMV and HHV-8 coinfection in CMV gastrointestinal diseases such as gastric ulcer and duodenitis of a renal transplant recipient. CMV serology tests before transplantation showed that the recipient was positive for CMV IgG and negative for IgM, and pre-transplant HHV-8 serology screening was not performed on the recipient or donor in this study. As there is a low seroprevalence of HHV-8 in the Republic of Korea and in Asia generally compared to other regions, HHV-8 serologic testing is not routinely performed in pre-transplant donor and recipient screening [4]. Therefore, coinfection of HHV-8 was not expected. CMV disease can be predicted following the detection of gastric ulcers and accompanying postural epigastric pain in transplant patients, although the morphological characteristic of gastric ulcers associated with CMV infection is non-specific [9–11].

To the best of our knowledge, no prior studies have reported a similar clinical manifestation of CMV and HHV-8 coinfection as we observed in this patient. In previous cases, peptic ulcers were observed in cases of CMV disease affecting the gastrointestinal tract [10, 11]. Therefore, had we not tested for HHV-8, the negative HHV-6 and HHV-7 immunohistochemistry tests would have suggested that the gastric ulcer observed in our case was only related to CMV disease, which could be interpreted as monism.

The possibility of HHV-6 or HHV-7 coinfection with CMV in solid organ transplant patients is well-known [2]. In several studies of solid organ transplant patients, coinfection of CMV with HHV-6 or HHV-7 affected allograft dysfunction as well as the development and progression of CMV disease [2]. In our study, immunohistochemical staining was negative for CMV coinfection with HHV-6 or HHV-7, but positive for HHV-8. Immunohistochemical staining in our case showed negative results for other herpesviruses and was positive for only CMV and HHV-8. The subject patient is Korean and has no foreign travel history, meaning his HHV-8 was not obtained from intermediate or high endemic HHV-8 regions such as Mediterranean countries, Middle-Eastern countries, the Caribbean, and Africa [3]. A primary HHV-8 infection was unlikely as the patient did not have a fever, splenomegaly, a rash, lymphoid hyperplasia, or pancytopenia. As Korea is a HHV-8 low-prevalence region, the possibility of HHV-8 transmission through infected organ transplantation is low [3]. In addition, the absence of HHV-8 serology test results for the donor and recipient limit the investigation of the HHV-8’s origin. The patient’s HHV-8 infection is presumed to have originated through salivary or blood transmission or sexual intercourse, and it was considered to be a reactivation of a latent infection due to the patient’s immunosuppressed status after transplantation [12]. Ganciclovir has an antiviral effect on HHV-8 and HHV-8 viremia did not develop when antiviral prophylaxis was administered with ganciclovir to solid organ transplant patients [13, 14]. Therefore, ganciclovir administered to this patient showed an antiviral therapeutic effect not only on CMV but also on HHV-8, suggesting that the clinical course of gastric ulcer and duodenitis related to coinfection of CMV and HHV-8 was improved.

There have been fewer studies on the relationship between CMV and HHV-8 in solid organ transplant patients compared with CMV and HHV-6 or HHV-7. A study of DNA level measurements of CMV and HHV-8 in peripheral blood monocytic cells of AIDS patients revealed that HHV-8 load was not predictive of CMV disease, and CMV load was not associated with KS [15]. However, CMV and HHV-8 have similar tissue tropism, and CMV coinfection was reported in the KS lesions of HIV positive and HIV negative patients [7, 16]. Coinfection with CMV is also known to activate HHV-8 lytic replication in a variety of cells including endothelial cells, fibroblasts, and keratinocytes [8]. Therefore, a mutual interaction related to CMV/HHV-8 coinfection is likely to exist in vivo, although the impact of these coinfecting pathogens on associated pathologies is not well-understood [6]. We did not evaluate the viral activity of HHV-8 because we did not measure HHV-8 serology and PCR monitoring for HHV8 viremia or HHV-8 plasma DNA level. However, in this study, HHV-8 was a more important determinant of CMV disease than detection of bystander activation or false-positive results because the patient had gastric ulcers and duodenitis with postural epigastric pain, HHV-8 was identified in immunohistochemical staining, and the patient’s immunosuppressed state after transplantation contributed to the reactivation of the two viruses and the progression of associated pathologies [5, 17]. In the future, additional studies are required on the prevalence of CMV/HHV-8 coinfection in solid organ transplant patients, the pathogenicity of CMV/HHV-8 viral interaction, and the effect on allograft.

Gastrointestinal symptoms associated with HHV-8 infection are generally observed in response to KS, and not as a consequence of the primary infection [5]. Post-transplant KS is the most commonly reported HHV-8-related disease and neoplastic manifestation after solid organ transplant [4]. The incidence of KS in renal transplant recipients is between 0.5% and 5% [18]. Although KS mainly occurs in the skin, the lymph nodes, gastrointestinal tract, liver, and lungs may also be involved [18]. This visceral involvement occurs in 10% of post-transplant KS cases, but KS involving the gastrointestinal tract appears to be particularly rare [4, 19, 20]. KS can invade the entire gastrointestinal tract and its clinical manifestations vary from asymptomatic to nausea, hemorrhage, perforation, and obstruction syndrome due to tumoral compression [21]. Gastrointestinal KS lesions have a variable appearance during an endoscopic examination, as ulcerated, flat, polypoid/nodular, and volcano-like lesions have been found [22]. Our patient did not present with a clinical pattern consistent with primary infection, and KS was excluded from the histopathologic findings. However, postural epigastric pain was present, a gastric ulcer was observed endoscopically, and the immunohistochemistry results were positive for HHV-8 in the gastric ulcer lesion. Even if the gastric ulcer observed in our patient was not a type of KS, which can be caused by the reactivation of HHV-8, HHV-8 may have contributed to his gastrointestinal disease and caused his gastrointestinal symptoms.

In conclusion, we reported the first case of gastric ulcers and duodenitis associated with a coinfection of CMV and HHV-8 after renal transplantation. In general, serologic evaluation of HHV-8 before transplantation is not recommended, however clinicians should consider HHV-8 as a potential source of coinfection with CMV in tissue-invasive CMV disease of renal transplant recipients.

## Data Availability

The data that support the findings of this study ara available from the corresponding author upon reasonable request.

## Abbreviations

CMV: Cytomegalovirus
HHV6: Human herpesvirus-6
HHV7: Human herpesvirus-7
HHV-8: Human herpesvirus-8
AIDS: Acquired Immune Deficiency Syndrome
KS: Kaposi’s sarcoma
HSV-1: Herpes Simplex Virus-1
EBV: Epstein-Barr Virus
VZV: Varicella Zoster Virus
HIV: Human Immunodeficiency Virus
HBV: Hepatitis B Virus
HCV: Hepatitis C Virus
